# Risk of epilepsy following first unprovoked and acute seizures: Cohort study

**DOI:** 10.1111/epi.18276

**Published:** 2025-02-03

**Authors:** Isaac J. Egesa, Symon M. Kariuki, Collins Kipkoech, Charles R. J. C. Newton

**Affiliations:** ^1^ Neuroscience Unit Kenya Medical Research Institute–Wellcome Trust Research Programme Kilifi Kenya; ^2^ Department of Health Data Science, Institute of Population Health Liverpool University Liverpool UK; ^3^ African Population and Health Research Centre Nairobi Kenya; ^4^ Department of Public Health Pwani University Kilifi Kenya; ^5^ Department of Psychiatry University of Oxford Oxford UK

**Keywords:** acute seizures, Africa, epilepsy, first unprovoked seizures, population attributable risk

## Abstract

**Objective:**

First unprovoked seizures and acute seizures are common and can develop into epilepsy. The risk of epilepsy following these seizures in community samples is not well established, and it is unclear whether the probability of subsequent unprovoked seizures following these seizures reaches the International League Against Epilepsy's threshold of 60%.

**Methods:**

We followed participants initially classified as having first unprovoked seizures, having acute seizures, or without seizures in a community‐based survey conducted in 2003 to estimate the subsequent risk of epilepsy in 2008 and 2021. The diagnosis of epilepsy in 2008 and 2021 was based on data from a community survey and health care visits to Kilifi County Hospital and the epilepsy clinic. Poisson regression models were used to compute incident risk ratios (IRRs) for epilepsy and population‐attributable risk (PAR); population‐attributable risk fractions (PAFs) were computed from contingency tables.

**Results:**

In the 5‐year follow‐up (censored in 2008 survey), the IRR for epilepsy was 23.3 (95% confidence interval [CI] = 14.2–38.2) for first unprovoked seizures and 10.4 (95% CI = 5.6–19.5) for acute seizures compared to the no‐seizure group. By 2021 (including 2008), the IRR was 18.4 (95% CI = 11.9–28.5) for first unprovoked seizures and 7.9 (95% CI = 4.3–14.5) for acute seizures compared to the no‐seizure group. The PAR for first unprovoked seizures and acute seizures was 29.0 and 8.0/1000 persons in the long‐term follow‐up. The PAF was 56.3% for first unprovoked seizures and 26.3% for acute seizures in the long‐term follow‐up. There was a high probability that a person with acute seizures (72%) or first unprovoked seizures (92%) developed epilepsy earlier than a person from the comparison group.

**Significance:**

First unprovoked seizures and acute seizures are associated with high risk for developing epilepsy. Neurological correlates for epilepsy risk following first unprovoked seizures should be investigated to inform epilepsy diagnosis and treatment.

**Key points**

- People with first unprovoked seizures and acute seizures have a significantly higher risk of developing epilepsy in rural Kenya.
- The probability that a person with acute seizures or first unprovoked seizures developed epilepsy earlier than a control exceeded the ILAE's 60% threshold for epilepsy diagnosis.
- First unprovoked seizures and acute seizures contribute a substantial burden of epilepsy, and the neurological correlates for this should be identified.
- Early identification and management of first unprovoked seizures and acute seizures will ameliorate epilepsy burden.

## 1 INTRODUCTION

Seizure disorders are very common, with an individual lifetime risk of 8%–10%.^1^ Acute seizures are the most common seizure disorders in children aged ≤6 years, and are known as febrile seizures when provoked by fever from nonneurological illnesses, or acute symptomatic seizures when in close temporal association with an acute central nervous system insult such as neurological febrile infection and metabolic perturbations.^2^ Unprovoked seizures suggest an enduring neurological predisposition to epilepsy, especially when at least two of these occur 24 h apart.^3^ First unprovoked seizures affect approximately 56–204 cases per 100 000 persons annually^4^, ^5^ and acute seizures affect 2%–5% of the child population.^6^ During the first years of life, the incidence of first unprovoked seizures is as high as 130 per 100 000 person‐years.^7^ In low‐ and middle‐income countries (LMICs), the burden of seizure disorders is greater than in other regions.^8^ Population‐based studies of all seizure disorders are limited, with acute seizures examined in a few studies in LMICs.^6^

Most of the available evidence on risk of epilepsy following first unprovoked seizures is based on studies from high‐income countries (HICs), which may not be representative of the situation in LMICs. The risk of recurrence of another seizure after first unprovoked seizures in hospital‐based settings from the United States and Brazil ranged from 27% to 71%,^9^, ^10^ depending on the study design, duration of follow‐up, risk factors in different populations, and populations studied. For example, children from the United States had higher risks of recurrence of unprovoked seizures and acute seizures than adults.^7^ Additionally, symptomatic etiology, focal seizures, neurodevelopmental delay, multiple seizures within 24 h, and family history of epilepsy were significant predictors of developing epilepsy after first unprovoked seizures among Portuguese children.^11^ These risk factors for the development of epilepsy following first unprovoked seizures are common in LMICs.^12^

There are few population‐based studies on the risk of epilepsy following first unprovoked seizures and acute seizures, yet understanding the risk of epilepsy after first unprovoked seizures and acute seizures is important in establishing the natural history of the disease to inform definition of individuals at risk of developing epilepsy and making treatment decisions. Also, a 60% chance of developing further unprovoked seizures after a first unprovoked seizures is one of the criteria introduced by the International League Against Epilepsy (ILAE) for the definition of epilepsy in 2014.^3^ Available studies from both HICs and LMICs are fraught with some limitations; for example, the risk of recurrence is not examined in population‐representative studies.^9^, ^13^ Some studies follow up risk of recurrence for first unprovoked seizures or acute seizures without a comparison group,^14^ and others introduce selection bias by basing follow‐ups on participants with elevated risks for epilepsy such as abnormal electroencephalograms (EEGs) and structural etiology.^15^

In LMICs, the risk of recurrent unprovoked seizures following acute seizures or first unprovoked seizures may be unreliable because follow‐up is often restricted to hospital samples.^16^ Also, seizures with fever are synonymously referred to as febrile seizures or acute symptomatic seizures, yet risks for epilepsy following each may be different.^16^ There is a paucity of evidence on contribution of first unprovoked seizures and acute seizures in the development of epilepsy in community‐based settings of Africa, where shared risk environmental factors such as central nervous system (CNS) infections^17^ and genetic sceptibility for seizures^18^ may be important. Understanding the risk of epilepsy following first unprovoked seizures and acute seizures in community settings of Africa may inform considerations for early initiation of prophylactic antiseizure medication (ASM) in those with these seizures to improve outcomes.

The population‐based studies of epilepsy and other seizures conducted in the rural settings of the Kenyan coast in 2003 and 2008^19^, ^20^ had cohorts of people with first unprovoked seizures and acute seizures who can be followed up to examine the short‐term risk (those with epilepsy in the 2008 survey and not the 2003 survey) and long‐term risk of epilepsy (those treated for epilepsy in a referral hospital in 2021 [censor date for this analysis] who did not have epilepsy in the 2003 survey).^21^ These studies were conducted in the Kilifi Health and Demographic Surveillance System (KHDSS), which links information of participants to a hospital database using unique personal identification.^22^ Using such population‐based cohorts of epilepsy, linked with hospital data from KHDSS, the downstream risk of epilepsy following first unprovoked seizures or acute seizures can be quantified through incident rate ratio (IRR), population‐attributable risk (PAR), and population‐attributable risk fraction (PAF). IRR is the incidence rate of epilepsy in the exposed group divided by the incidence rate in the unexposed group and can be used to approximate probabilities of occurrence using the formula IRR / (1 + IRR); probability is the extent to which a randomly chosen participant from the cases will develop epilepsy first compared to a randomly selected participant from the comparison group, if both did not have an event at baseline. PAR is the difference in the risk of epilepsy between the total population (both exposed and unexposed group) and the unexposed group, whereas PAF is the proportion of cases for an outcome that can be attributed to a certain risk factor, and is expressed as PAR divided by the risk in the total population.^23^

In this study, we examined the short‐term and long‐term risk of developing epilepsy among individuals with first unprovoked seizures and acute seizures compared to those without seizures in a population‐based setting, determining whether the probability of a person from the exposed group developing subsequent unprovoked seizures earlier than that from the comparison group was >60%, the epilepsy diagnosis threshold set by the ILAE following first unprovoked seizures. Specifically, we computed the cumulative incidence, IRR, PAR, and PAF of epilepsy associated with first unprovoked seizures or acute seizures.

## 2 MATERIALS AND METHODS

### 2.1 Study setting and study population

The study was conducted in a defined rural area on the Kenyan coast, called KHDSS, covering a population of approximately 300 000 people. KHDSS was established in the year 2000 as a record of births, pregnancies, migration of residents in and out of the area, and deaths, and it undergoes regular census three times every year.^22^ Most residents are Mijikenda speakers and are poor, with inadequate access to proper sanitation and low literacy levels. The main economic activity in this region is fishing and subsistence farming. Residents of the KHDSS are assigned a unique identification number, which enables data from hospital admissions to be individually linked to the census data. There is one major hospital (Kilifi County Hospital [KCH]) for admission of patients that draws most of its admissions from the KHDSS. The KCH records all admissions in the database and codes diagnoses according to the International Classification of Diseases 10.

The Epilepsy and Neurodevelopment Clinic was established by the Neuroscience Unit of the KEMRI–Wellcome Trust Research Programme in collaboration with KCH to provide care services to people with epilepsy identified from earlier community‐based surveys of neurological impairments.^24^, ^25^ The clinic serves residents of KHDSS and other neighboring counties of Mombasa, Kwale, Tana River, and Lamu, and epilepsy referrals from other hospitals in the region. The epilepsy clinic is run by experienced epilepsy clinicians, researchers, psychologists, and a neurologist. The clinic offers EEG services for classification of seizure semiology in epilepsy, and for ruling out other paroxysmal events, for example, psychogenic nonepileptic seizures. Patients with epilepsy visiting the epilepsy clinic had access to free ASM. Those admitted to KCH during the analysis period had access to basic laboratory investigations (e.g., full blood count and blood smear for malaria diagnosis); neuroimaging services were only available at private health services at a cost. There were no specialist epilepsy services in the study area around 2003 when the first epilepsy survey was conducted,^19^ but the epilepsy clinic was set up thereafter to support the epilepsy survey conducted in 2008.^20^ Before the epilepsy clinic was set up, patients were seen through the general outpatient and inpatient services of KCH. The number of people with epilepsy visiting the epilepsy clinic has increased significantly over the years, necessitating initiation of task‐sharing interventions in 2019 to strengthen capacity to detect and manage epilepsy in the primary health care facilities located within the KHDSS.

### 2.2 Cohort identification

Between August and November 2003, a large population‐based epilepsy cross‐sectional epidemiological survey was conducted within KHDSS to identify persons with active convulsive epilepsy (ACE). Details of this epidemiological survey are described elsewhere.^19^ Briefly, in stage I, the head of each household was asked two screening questions on convulsions by the KHDSS team during a door‐to‐door census. In stage II, conducted in August and November 2003, trained fieldworkers administered a detailed standardized questionnaire (Appendix S1) to positive respondents in stage I.^19^ Individuals suspected of having epilepsy in stage II were invited for clinical evaluation, where a detailed clinical history was taken by clinicians trained in epilepsy and fluent in the local language. Epilepsy was defined according to the ILAE definition as a history of two unprovoked seizures occurring 24 h apart.^3^ In this 2003 cross‐sectional epilepsy survey, we identified a baseline cohort of people with first unprovoked seizures and acute seizures and a comparison group of individuals without seizures in stage II of the survey. Even if participants aged < 6 years were excluded in determination of epilepsy estimates at stages II and III of the 2003 survey, those aged 6 years and older were asked to recall seizure history in their first 6 years, including first unprovoked seizures and acute seizures, which are common in this age period. The data on acute seizures, used in the present analysis, were not included in epilepsy data published previously.^19^, ^20^ In the present analysis, we excluded participants with a known diagnosis of epilepsy at the baseline from the cohort to be followed up.

### 2.3 Follow‐up for risk of epilepsy

**FIGURE 1:**
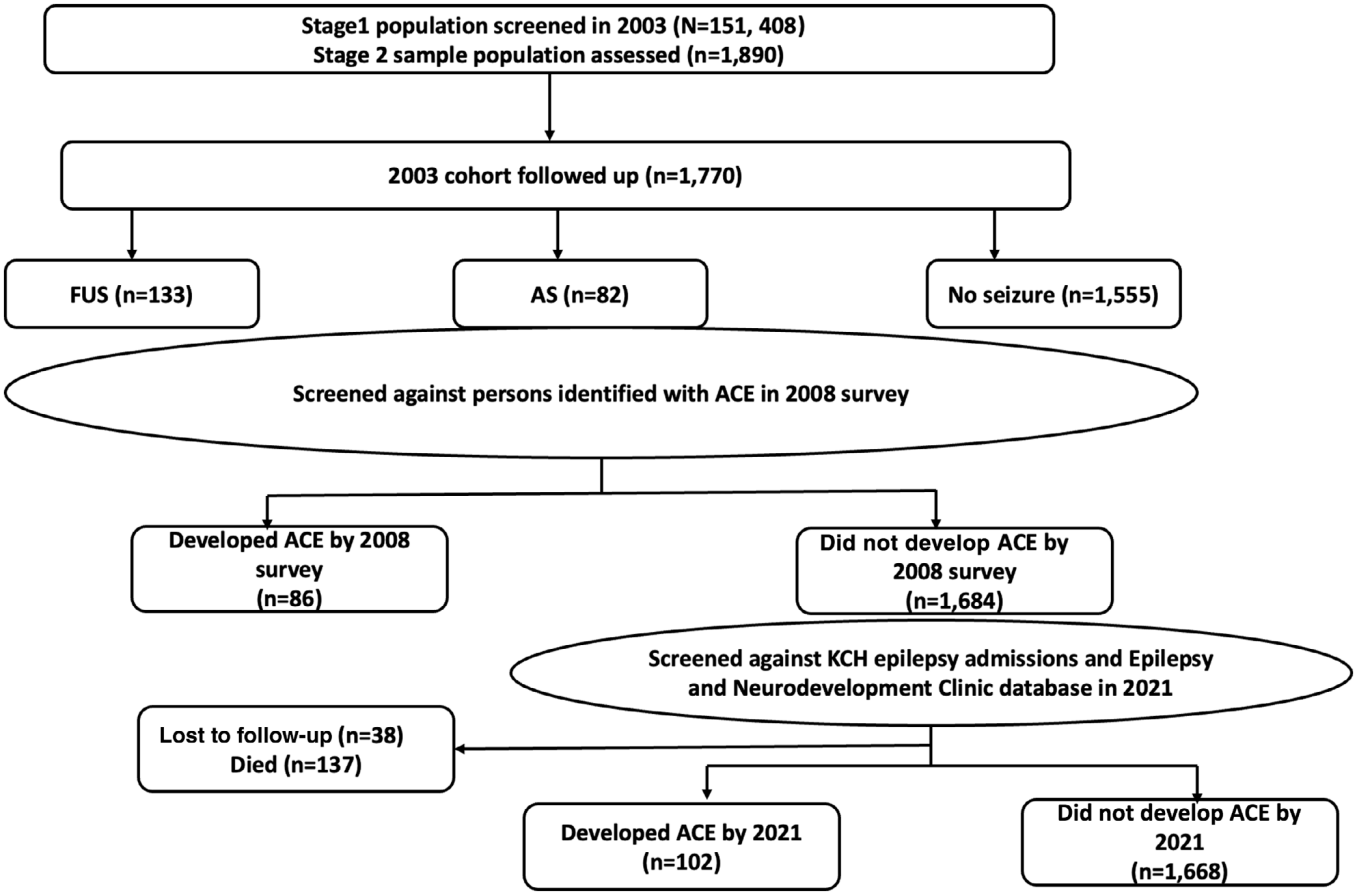
Cohort identification and follow‐up process. The baseline cohorts were drawn from the 2003 community‐based epilepsy survey, whereas the epilepsy diagnosis used in follow‐ups was drawn from a community‐based epilepsy survey in 2008 and epilepsy care visits to hospital and clinics until 2021. ACE, active convulsive epilepsy; AS, acute seizures; FUS, first unprovoked seizures; KCH, Kilifi County Hospital.

First, a unique personal identifier was used to link individuals with first unprovoked seizures, those with acute seizures, and those with no history of seizures in the 2003 survey against persons identified with epilepsy in a 2008 community‐based survey.^20^ Those who matched with people with epilepsy identified in 2008 were considered as new or incident cases of epilepsy within 5 years. A further follow‐up was made on the remaining first unprovoked seizures and acute seizures and those with no history of seizures up to April 2021 by searching the KCH epilepsy admission register and Epilepsy and Neurodevelopment Clinic database^21^ for additional new cases of epilepsy using a unique personal identifier. Search terms “epilepsy” or “epileptic fits” or “convulsions” were run in the KCH admissions database, and medical records were reviewed to identify persons who fulfilled a diagnosis of epilepsy of two unprovoked seizures. Those who matched in the second follow‐up together with first follow‐up cases were considered new or incident cases of epilepsy within 18 years (Figure 1). Of the 1890 study participants screened for first unprovoked seizures and acute seizures in stage II, 1770 (93.7%) were successfully linked to the 2008 epilepsy and to the epilepsy care services at KCH and the epilepsy clinic.

### 2.4 Definition of terms

ACE was defined based on two or more unprovoked seizures (tonic and/or clonic seizures) occurring 24 h apart, with at least one seizure episode in the past 12 months before the survey.^3^ A new case of epilepsy was defined as participants who were found not to have ACE at the baseline (the 2003 community‐based survey) and developed epilepsy during the follow‐up period. Acute seizures were defined as seizures associated with fever or an acute illness or CNS insult.^2^ A first unprovoked seizure was one episode of seizure within 24 h with no immediate underlying cause.^3^ We defined the comparison group as people who screened negative for ACE in the 2003 survey and did not meet the definition of acute seizures or first unprovoked seizures on the baseline survey.

### 2.5 Statistical analysis

Statistical analyses were performed using Stata version 18 (Stata Corporation) and R statistical software. We used descriptive statistics such as mean, median, SD, and percentiles to report continuous variables. The primary outcome was the development of epilepsy defined as two or more unprovoked seizures occurring 24 h apart. We compared the absolute risk of epilepsy among first unprovoked seizures, acute seizures, and the comparison group. The IRR of developing epilepsy and 95% confidence interval (CI) were calculated by estimating a Poisson regression model with exposure status (first unprovoked seizures and acute seizures), age, and sex as predictor variables and epilepsy incidence as a response variable. The Poisson model was assessed for misspecification and robustness using diagnostic plots and *linktest* in Stata. The attributable risk percent and PAR for first unprovoked seizures and acute seizures were calculated using the formulae (*r*1 − *r*0) / *r*1 × 100 and *r* − *r*0, respectively,^26^ whereas PAF was estimated using the formula PAR/*r*,^27^ where *r*1 is the risk of epilepsy in the exposed group (first unprovoked seizures or acute seizures), *r*0 is the risk of epilepsy in those without seizures (controls), and *r* is the risk of epilepsy in the study population at different censoring stages. The probability risk of subsequent development of epilepsy following exposure to first unprovoked seizures and acute seizures was calculated as follows: $\frac{\mathrm{IRR}}{1+\mathrm{IRR}}$.^6^, ^28^ Pearson chi‐squared test was used to compare the frequency differences of categorical variables between groups. A one‐way analysis of variance statistical test was used to compare the difference among more than two groups, and casewise analysis was used due to very low attrition.

### 2.6 Ethical approval

Written informed consent was obtained from adult participants and assent from children younger than 18 years. Permission to conduct the study was approved by the Kenya Medical Research Institute (KEMRI), Scientific Ethics Review Unit (SERU; KEMRI/SERU/CGMR‐C/125/3701).

## 3 RESULTS

### 3.1 Sociodemographic characteristics

At total of 1770 individuals screened in stage II of the 2003 survey, who did not meet the ILAE definition of epilepsy, were initially classified as first unprovoked seizures (*n* = 133, 7.5%), acute seizures (*n* = 82, 4.6%), or not having a seizure (*n* = 1555, 87.8%) and were successfully followed (Figure 1). The median age of the cohort was 11 years (interquartile range [IQR] = 8–18), and half of the participants were between 6 and 12 years old (Table 1). The first unprovoked seizures, acute seizures, and comparison groups (those without seizures) had a median age of 17 years (IQR = 12–25), 10.5 years (IQR = 8–18), and 11 years (IQR = 8–16), respectively, with significant differences in age between groups (*p* < .01). In summary, 51% of the cohort were male, with no difference between the first unprovoked seizures, acute seizures, and comparison groups (*p* = .916). The distribution of participants successfully followed to the end was significantly higher among the comparison group compared to first unprovoked seizures and acute seizures (*p* < .01).

**TABLE 1:** Sociodemographic characteristics of study participants.

Characteristics	No‐seizure group, *n* = 1555	Acute seizures, *n* = 82	Single unprovoked seizures, *n* = 133	Overall, *N* = 1770
Sex, *n* (%)				
Male	795 (51.1)	43 (52.4)	66 (49.6)	904 (51.1)
Female	760 (48.9)	39 (47.6)	67 (50.4)	866 (48.9)
Vital status by 2021, *n* (%)				
Alive	1424 (91.6)	66 (80.5)	105 (78.9)	1595 (90.1)
Died	98 (6.3)	12 (14.6)	27 (20.3)	137 (7.7)
Lost to follow‐up	33 (2.1)	1 (1.2)	1 (.7)	38 (2.2)
Age, years, median (interquartile range)	11 (8–16)	10.5 (8–18)	17 (12–25)	11 (8–17)
Age groups in years, *n* (%)				
6–12	939 (60.4)	53 (64.6)	39 (29.3)	1031 (58.3)
13–18	280 (18.0)	9 (10.9)	34 (25.6)	323 (18.3)
19–28	127 (8.2)	12 (14.6)	34 (25.6)	173 (9.8)
29–49	115 (7.4)	6 (7.3)	19 (14.3)	140 (7.9)
>50	94 (6.1)	2 (2.4)	7 (5.3)	103 (5.8)

### 3.2 Short‐term risk of epilepsy (5 years)

Among participants of the 2003 cohort, 4.8% of persons developed epilepsy by 2008. The absolute risk of developing epilepsy was highest at ages between 6 and 12 years, with a significant difference in terms of distribution across age groups (*p* < .01). The absolute risk of epilepsy was higher among the first unprovoked seizures cohort (35%, 95% CI = 27–44) compared to the acute seizures (17%, 95% CI = 9–27) and no‐seizure groups (1.6%, 95% CI = 1.0–2.3) in the 5‐year follow‐up (*p* < .0001; Table 2).

**TABLE 2:** Short‐term and long‐term risk for occurrence of epilepsy following acute seizures and first unprovoked seizures.

	No‐seizure group (95% CI)	Acute seizures (95% CI)	First unprovoked seizures (95% CI)
Short‐term risk of epilepsy censored in 2008 [5 years]			
Epilepsy diagnosis, *n* (%)	25 (1.6)	14 (17.1)	47 (35.3)
Absolute risk	1.6 (1.0–2.3)	17.1 (9–27)	35.3 (27–44)
IRR^a^	Reference	10.4 (5.6–19.5)	23.3 (14.2–38.2)
AR	Reference	.15 (.07–.24)	.34 (.26–.42)
AR %	Reference	90.6 (81.8–99.3)	95.5 (86.7–104.2)
PAR per 1000	Reference	7.7 (.3–15.1)	26.6 (16.9–36.2)
PAF %	Reference	32.5 (1.5–63.5)	62.3 (39.7–84.9)
Probability	Reference	80 (71–86)	93 (88–97)
Long‐term risk of epilepsy censored in 2021 [18 years]^b^			
Epilepsy diagnosis, *n* (%)	35 (2.3)	15 (18.3)	52 (39.1)
Absolute risk	2.2 (1.6–3.1)	18 (10–28)	39 (30–47)
IRR^a^	Reference	7.9 (4.3–14.5)	18.4 (11.9–28.5)
AR	Reference	.16 (.06–.26)	.37 (.28–.45)
AR %	Reference	87 (78.6–96.8)	94.2 (85.2–103.3)
PAR per 1000	Reference	8.0 (−.3 to 16.3)	29.0 (18.5–39.9)
PAF %	Reference	26.3 (−1.0–53.6)	56.3 (35.9–76.8)
Probability	Reference	78 (68–84)	92 (85–95)

*Note:* AR % = (*r*1 − *r*0) / *r*1, PAR = *r* − *r*0, PAF = PAR/*r* × 100, probability risk = IRR/1 + IRR; where *r*1 is the proportion of epilepsy in the exposed group (acute seizures or first unprovoked seizures), *r*0 is the proportion of epilepsy in those without seizures, and *r* is the proportion of all epilepsy cases in the sample population followed.

Abbreviations: AR, attributable risk; CI, confidence interval; IRR, incident rate ratio; PAF, population‐attributable risk fraction; PAR, population‐attributable risk.

^a^ Adjusted for age and sex.

^b^ The long‐term risks and probabilities reported in 2021 represent total cumulative risks and probabilities from 2003 to 2021.

First unprovoked seizures and acute seizures had a 23‐fold (IRR = 23.3, 95% CI = 14.2–38.2) and 10‐fold (IRR = 10.4, 95% CI = 5.6–19.5) higher epilepsy incidence, respectively, compared to the no‐seizure group in the 5‐year follow‐up, after adjusting for age and sex. The risk difference between first unprovoked seizures and acute seizures compared to the no‐seizure group was 95% (95% CI = 86–104) and 90% (95% CI = 81–99), respectively. The PAR of epilepsy was 26 per 1000 person (95% CI = 17–36 per 1000) for first unprovoked seizures and 7.7 per 1000 person (95% CI = .3–15 per 1000) for acute seizures. The PAF for epilepsy associated with first unprovoked seizures was 62.3% (95% CI = 39–84) and with acute seizures was 32.5% (95% CI = 1.5–63.5). The 5‐year probability that a person with first unprovoked seizures and with acute seizures developed epilepsy earlier than a person from the comparison group was 93% and 80%, respectively (Table 2).

### 3.3 Long‐term risk of epilepsy (18 years)

**FIGURE 2:**
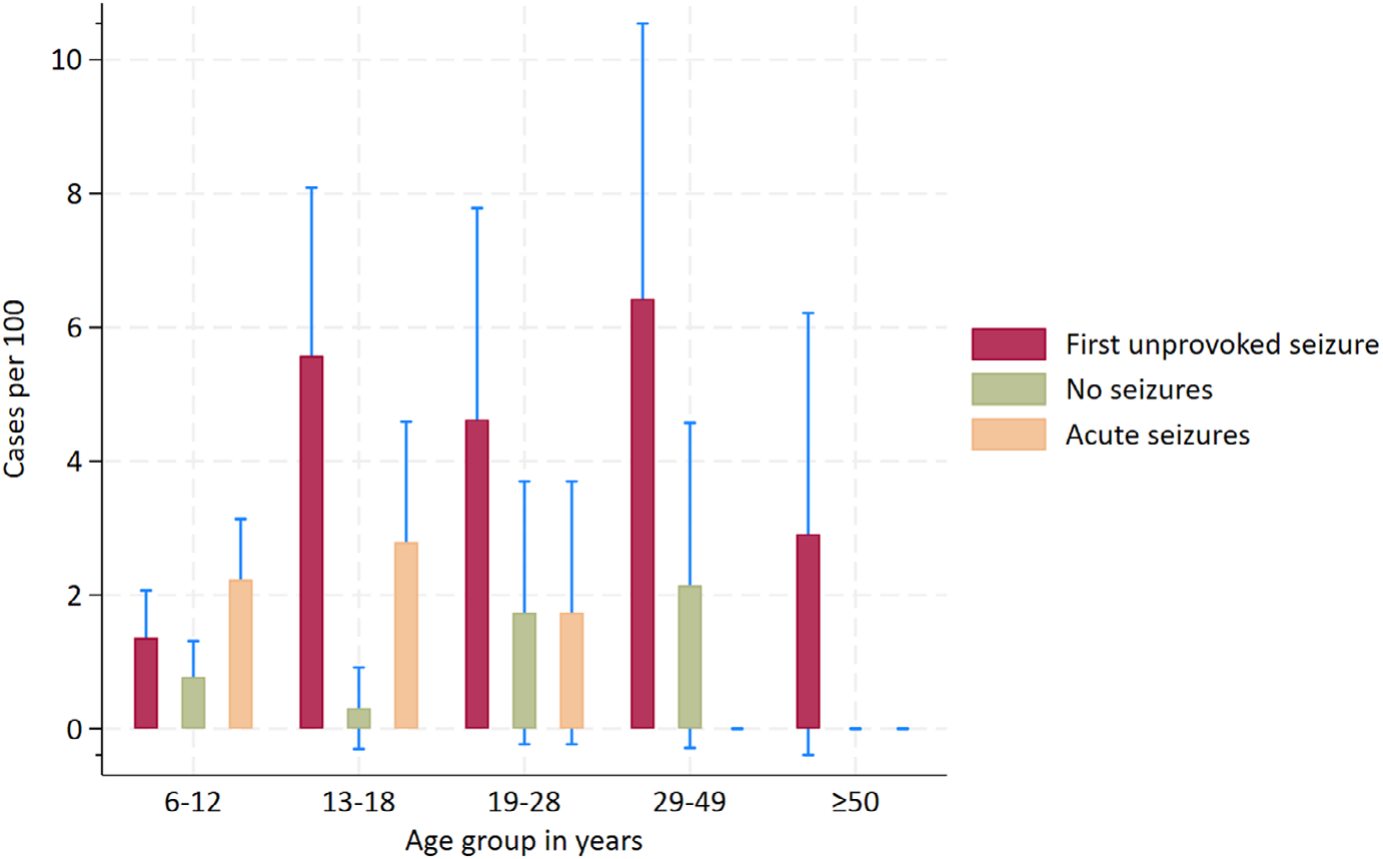
Distribution of epilepsy incidence in exposure groups by 2021. The frequency of epilepsy per 100 across the follow‐up periods is provided by exposure group (first unprovoked seizures, acute seizures, and comparison group) and by age group.

In the long‐term follow‐up, 5.7% of the sample population developed epilepsy. The cumulative incidence of epilepsy in the first unprovoked seizures and acute seizures groups was significantly higher across all age groups compared to the no‐seizure group (Figure 2). Participants aged 13–49 years had the highest epilepsy incidence in the first unprovoked seizures group, whereas those aged 19–49 years had the highest epilepsy incidence in the acute seizures group. The long‐term absolute risk of epilepsy was 39% (95% CI = 30–47) for first unprovoked seizures, 18% (95% CI = 10–28) for acute seizures, and 2.2% (95% CI = 1.6–3.1) for the no‐seizure group.

Participants who had a first unprovoked seizure in 2003 were found to have an 18.4‐fold higher epilepsy incidence (95% CI = 11.9–28.5) compared to the no‐seizure group. Similarly, the IRR for acute seizures was 7.9 (95% CI = 4.3–14.5). The epilepsy risk difference was consistent with the short‐term risk at 94.2% (95% CI = 85.2–103.3) for first unprovoked seizures and 87.0% (95% CI = 78.6–96.8) for acute seizures compared to the no‐seizure group. The long‐term PAR of epilepsy associated with first unprovoked seizures was 29 per 1000 persons (95% CI = 35.9–76 per 1000), whereas that for acute seizures was 8.0 per 1000 persons (95% CI = −.3 to 16.3 per 1000). The PAF showed that approximately 56.3% (95% CI = 35.9–76.8) and 26.3% (95% CI = −1.0 to 53.6) of epilepsy diagnoses can be attributed to exposure to first unprovoked seizures and acute seizures, respectively. Lastly, the probability that a person with first unprovoked seizures and acute seizures developed epilepsy earlier than a person from a comparison group remained high (Table 2).

## 4 DISCUSSION

In this study, the short‐term epilepsy risk (censored in 2008) and the long‐term epilepsy risk (censored in 2021) were significantly higher in the first unprovoked seizures group and acute seizures group compared to the no‐seizure group, suggesting a differential underlying risk for subsequent unprovoked seizures in the cohorts. The IRR of epilepsy was consistently greater in first unprovoked seizures than acute seizures during the two follow‐up periods when compared with the no‐seizure group, implying greater propensity for development of epilepsy in the first unprovoked seizures group. The PAR for epilepsy was 26.6 per 1000 persons for first unprovoked seizures and 7.7 per 1000 persons for acute seizures and was not statistically different from the long‐term estimates. Similarly, the PAF for epilepsy implies that a substantial proportion of epilepsy cases in the community are related to first unprovoked seizures (up to 62.3%) or acute seizures (up to 32.5%), depending on the duration of follow‐up. The probability that a person with first unprovoked seizures or acute seizures developed epilepsy earlier than a person from the comparison group exceeded the ILAE general recurrence threshold of 60% recommended for definition of epilepsy after a first unprovoked seizure.^3^

These findings suggest a substantial proportion of epilepsy cases in this study population can be avoided if measures to initiate prophylactic management (with ASM) of first unprovoked seizures and acute seizures are instituted. The 35% cumulative risk of epilepsy associated with first unprovoked seizures in a 5‐year follow‐up aligns with other studies finding risks of 30%–40%.^29^, ^30^ The marginal IRR reduction from short‐term to long‐term follow‐up in the first unprovoked seizures group is likely to be explained by loss to follow‐up and mortality. The high probability of developing epilepsy highlights the importance of early prophylactic intervention and close monitoring of individuals who experience these seizure types. Long‐term risk of epilepsy following prophylactic treatment for acute seizures is rarely examined.^31^ The finding that the probability of subsequent unprovoked seizures censored in 2008 and 2021 was high and satisfied the ILAE definition of epilepsy^3^ is noteworthy. However, neurological markers such as epileptiform discharges on the EEG, abnormal neurological examinations and neuroimaging features following first unprovoked seizures should be determined and used as proxies for predicting epilepsy risk and guiding prophylactic treatment.

Acute seizures posed a significant risk of epilepsy in this cohort, with a cumulative risk of 17% and an IRR of 10.4, higher than the 5% risk reported in previous hospital cohorts.^16^ After 2008, follow‐up was based on hospital data only, with varied durations of follow‐up, some <1 year. However, our results seem to be consistent with other research that showed a 10%–20% risk among people with complex acute seizures,^32^ including in UK cohorts of acute seizures.^33^ Long‐term follow‐up of epilepsy following acute seizures beyond 5 years needs rethinking, as there was no change in absolute risk and IRR at the 18‐year follow‐up.

The PAR of 26.6 per 1000 persons for first unprovoked seizures and 7.7 per 1000 persons in a 5‐year follow‐up indicates an absolute epilepsy risk difference of 26 and 7 cases per 1000 people, respectively, between the sample population and those not exposed to a seizure. These estimates were statistically similar for long‐term follow‐up. However, these findings should be interpreted with caution, as we did not have data to explore all important neurophysiological and medical correlates of higher epilepsy risks in the study.

The relatively high PAF for epilepsy following first unprovoked seizures and acute seizures have implications in prophylactic management interventions. However, the public health value of PAF should be interpreted carefully,^27^ because the associated estimates do not necessarily infer a causal relationship, as not all persons with a first unprovoked seizure develop subsequent unprovoked seizures, and development of epilepsy follows an interaction of many other risk factors including first unprovoked seizures. The causal assumptions may not hold in our case, because some of the first unprovoked seizures or acute seizures cases may have been missed epilepsy in the baseline survey if the respondents did not provide sufficient clinical information to substantiate a diagnosis. It is therefore possible that this would overestimate the PAF. The probabilities would also be high if there were fewer risks of epilepsy in the comparative group of those without epilepsy or seizures or if the screening tool had low specificity for ruling out false negatives. The risks for epilepsy also appeared to vary according to duration of follow‐up at least for first unprovoked seizures; the data did not support sensitivity analyses of other risk factors beyond age and sex. Nonetheless, identifying neurological correlates for supporting use of first unprovoked seizures in predicting epilepsy during community surveys should be prioritized, as this may reduce costs required for longitudinal follow‐up studies.

The decision to start ASM for persons with first unprovoked seizures is controversial.^34^, ^35^, ^36^ Current evidence shows that treatment of first unprovoked seizures should be based on an individualized assessment of the high risk of recurrence and the benefit of treatment supersedes the adverse effects. Prophylactic management of first unprovoked seizures and acute seizures can be prioritized in those with abnormal findings on neurological examination, neuroimaging, and EEG. Most studies on first unprovoked seizures are from HICs, with little known about the outcome of exploring treatment options for first unprovoked seizures and acute seizures in other settings. Appropriate health education and counseling should be considered in such settings. Public health interventions such as malaria prevention and immunization against tropical diseases should be reinforced to reduce acute seizure incidence^37^ and subsequent epilepsy risk.^38^ Training primary health care professionals on early identification of first unprovoked seizures is needed to initiate early counseling and recognize recurrence to avert the economic burden of epilepsy care.

### 4.1 Strengths and limitations

The study demonstrates a significant risk of epilepsy following first unprovoked seizures and acute seizures. The large sample size and low attrition rate ensured precise and powered outcomes, supported by a robust demographic surveillance system. We employed integrated follow‐ups through the hospital, survey, and clinic covering all possible incidences of epilepsy. Because similar risk factors for epilepsy may be reported differentially across the three study groups, including a nonseizure group allows testing of the hypothesis that future risk for subsequent unprovoked seizures would be large in those who have already experienced first unprovoked seizures and acute seizures. However, the study was limited to passive surveillance only at two cross‐sectional endpoints and did not focus on nonconvulsive seizures. If some nonconvulsive seizures were missed in those with first unprovoked seizures at baseline, this may inflate the epilepsy risks during follow‐up. During the cross‐sectional surveys that generated the cohorts for follow‐up, there was no capacity to determine different underlying causes and pathophysiological mechanisms of febrile seizures and acute symptomatic seizures, which can impact their ouctomes.^6^ The long‐term follow‐up leveraged hospital data, which may exclude a proportion of cases in the community due to stigma or if these were perceived less severe for treatment. Future studies should assess multiple time points and document all seizure types. There was no systematic collection of medical history and neuroinvestigation, for example, magnetic resonance imaging, for those with first unprovoked seizures or acute seizures to determine risk factors associated with subsequent risk. Also, lost follow‐ups were not assessed for epilepsy development due to logistics and cost, and past seizure events may be subject to recall bias, potentially underestimating epilepsy risk.

## 5 CONCLUSIONS

Exposure to first unprovoked seizures and acute seizures significantly increases the risk of developing epilepsy compared to individuals with no seizure history. The probability that this recurrence occurs earlier in those with first unprovoked seizures or acute seizures compared to controls is higher than the 60% general recurrence threshold suggested by the ILAE, but future risk correlates such as abnormal findings on neurological examinations, neuroimaging, and EEG should be identified. In settings with high‐risk epilepsy associated with first unprovoked seizures and acute seizures, appropriate prophylactic management interventions and counseling should be considered, including possible initiation of ASM.

## AUTHOR CONTRIBUTIONS

Isaac J. Egesa, Symon M. Kariuki, and Charles R. J. C. Newton conceptualized and designed the study. Isaac J. Egesa, Symon M. Kariuki, Collins Kipkoech, and Charles R. J. C. Newton were responsible for data management, analysis, and choosing data visualization methods. Isaac J. Egesa and Symon M. Kariuki wrote the initial draft. All authors provided feedback on the initial draft and approved the manuscript for publication.

## CONFLICT OF INTEREST STATEMENT

The other authors report no disclosures. We confirm that we have read the Journal's position on issues involved in ethical publication and affirm that this report is consistent with those guidelines.

## Supporting information


Appendix S1

## Data Availability

The data that support the findings of this study are available on request from the corresponding author, through emailing the KEMRI‐Wellcome Trust Research Programme's Data Governance Committee at dgc@kemri‐wellcome.org. The data are not publicly available due to privacy or ethical restrictions.
