# Supplementary material for: Risk of epilepsy following first unprovoked and acute seizures: Cohort study
Source: Epilepsia. 2025 Feb 3;66(4):1223–33. doi: 10.1111/epi.18276 (PMC11997935; doi:10.1111/epi.18276)
Supplement: Supplementary file 1 — Appendix S1 [file EPI-66-1223-s001.docx]

**Appendix S1: Screening questionnaires**

1. Have you ever had a fit?
2. Has someone ever told you that you have fits?
3. Have you ever been told that you have epilepsy or epileptic fits?
4. Have you ever had attacks in which you fall to the ground with loss of consciousness?
5. Have you ever fallen to the ground without a reason and experienced?
6. Twitching?
7. Shaking of the arms or legs without control?
8. Wetting yourself?
9. Biting of the tongue?
10. Have you ever been told by a doctor that you have epilepsy or epileptic fits?
